# Ionizing radiation and melanism in Chornobyl tree frogs

**DOI:** 10.1111/eva.13476

**Published:** 2022-09-07

**Authors:** Pablo Burraco, Germán Orizaola

**Affiliations:** ^1^ Animal Ecology, Department of Ecology and Genetics, Evolutionary Biology Centre Uppsala University Uppsala Sweden; ^2^ Doñana Biological Station (CSIC) Seville Spain; ^3^ School of Biodiversity, One Health & Veterinary Medicine University of Glasgow Glasgow UK; ^4^ IMIB‐Biodiversity Research Institute (Univ. Oviedo‐CSIC‐Princip. Asturias) University of Oviedo Mieres Asturias Spain; ^5^ Zoology Unit, Department of Biology of Organisms and Systems University of Oviedo Oviedo Asturias Spain

**Keywords:** animal colouration, Chernobyl, environmental pollution, evolutionary physiology, melanism, oxidative stress, radioactivity

## Abstract

Human actions are altering ecosystems worldwide. Among human‐released pollutants, ionizing radiation arises as a rare but potentially devastating threat to natural systems. The Chornobyl accident (1986) represents the largest release of radioactive material to the environment. Our aim was to examine how exposure to radiation from the Chornobyl accident influences dorsal skin coloration of Eastern tree frog (*Hyla orientalis*) males sampled across a wide gradient of radioactive contamination in northern Ukraine. We assessed the relationship between skin frog coloration (which can act as a protective mechanism against ionizing radiation), radiation conditions and oxidative stress levels. Skin coloration was darker in localities closest to areas with high radiation levels at the time of the accident, whereas current radiation levels seemed not to influence skin coloration in Chornobyl tree frogs. Tree frogs living within the Chornobyl Exclusion Zone had a remarkably darker dorsal skin coloration than frogs from outside the Zone. The maintenance of dark skin coloration was not linked to physiological costs in terms of frog body condition or oxidative status, and we did not detect short‐term changes in frog coloration. Dark coloration is known to protect against different sources of radiation by neutralizing free radicals and reducing DNA damage, and, particularly melanin pigmentation has been proposed as a buffering mechanism against ionizing radiation. Our results suggest that exposure to high levels of ionizing radiation, likely at the time of the accident, may have been selected for darker coloration in Chornobyl tree frogs. Further studies are needed to determine the underlying mechanisms and evolutionary consequences of the patterns found here.

## 1 INTRODUCTION

Habitat destruction, climate change and pollution are key factors damaging natural systems nowadays (Rands et al., 2010). Pollutants, in particular, are widespread in nature and represent one of the more powerful forces of ecological and evolutionary change (Palumbi, 2001; Spurgeon et al., 2020). Strong selective factors can induce fast adaptive responses, and signs of rapid adaptation to some pollutants have been observed across many taxa (Nacci et al., 2016; Pitelka, 1988; Reid et al., 2016), although these responses are far from generalized (e.g. Brady et al., 2017; Loria et al., 2019). Among the large diversity of pollutants introduced into the environment, radioactive contamination caused by human activities generates broad societal and scientific concern (e.g. Beresford & Copplestone, 2011). The release of radionuclides from nuclear power plants, as occurred after the accidents in Chornobyl (Ukraine) in 1986, and Fukushima (Japan) in 2011, represents the most evident case of public concern about the impact that ionizing radiation can have on living organisms.

The accident at the Chornobyl nuclear power plant on the 26 April 1986 caused the largest release of radioactive material to the environment in human history (UNSCEAR, 1988). Hence, the Chornobyl area constitutes a key scenario for evaluating the eco‐evolutionary consequences of the exposure to ionizing radiation on wildlife. The exposure to extremely high levels of radiation after the Chornobyl accident caused rapid environmental damage (Beresford et al., 2016; Møller & Mousseau, 2006; Sazykina & Kryshev, 2006; Yablokov et al., 2009). Such acute exposure to radiation negatively affected the physiology, morphology and genomics of different species inhabiting the Chornobyl area (e.g. Kryshev et al., 2005; Møller & Mousseau, 2006, 2015, 2016; Yablokov et al., 2009). However, recent studies have reported the presence of large and diverse animal communities, a lack of negative effects of current levels of radiation in many taxa, and even signs of adaptation to radiation (e.g. Burraco, Bonzom, et al., 2021; Deryabina et al., 2015; Galván et al., 2014; Jernfors et al., 2018; Kovalchuk et al., 2004; Møller & Mousseau, 2016; Schlichting et al., 2019). Thus, more than three decades after the accident, there is still high scientific discrepancy about the long‐term effects of the accident on wildlife (Beresford et al., 2020).

Ionizing radiation is harmful because it can damage DNA and other biomolecules, causing cell malfunctions and increasing mortality risk (Alizadeh et al., 2015; Lehnert, 2007; Mothersill & Seymour, 2014). Different mechanisms, such as DNA damage repair pathways have been proposed as candidates that may facilitate life in radio‐contaminated environments. Organisms exposed to ionizing radiation can plastically upregulate the expression of these repair and protection mechanisms (e.g. Jernfors et al., 2018; Kesäniemi et al., 2019; Mustonen et al., 2018). However, these processes can be physiologically costly and unable to fully buffer the impact of radiation, which often results in deleterious mutations or lower individual performance. An alternative protection from ionizing radiation may come through changes in pigmentation. In animals, coloration plays a key role in several ecological functions such as sexual selection, defence from predators and health maintenance (Caro, 2005; Cuthill et al., 2017; Mackintosh, 2001). Previous studies have also revealed that melanin‐based coloration can mitigate the impact of different forms of radiation, including ionizing radiation (Cordero & Casadevall, 2020; Dadachova et al., 2007, 2008; Dadachova & Casadevall, 2008). However, whilst melanization has been showed to have a protective role against ionizing radiation in organisms with relatively simple level of organization, such as fungi, evidence suggests that costs of melanin‐based colorations may be often higher than benefits in wild vertebrates (Galván et al., 2011, 2014).

Amphibians occupy both the terrestrial and the aquatic environments during their life cycle and are, thus, exposed to a full range of radiation sources in contaminated environments. Furthermore, they often show low vagility and are highly philopatric to their natal ponds (Cayuela et al., 2020), which facilitates the estimation of their potential exposure to radiation and makes them ideal subjects for evaluating the effects of radioactive contamination on wild vertebrates. However, work on amphibians in radio‐contaminated environments is still scarce (Orizaola, 2022). Recent studies have reported, for example, that radiation exposure was linked to an increase in mutation rates and mitochondrial DNA damage in frogs from radio‐contaminated areas (Car et al., 2022; Gombeau et al., 2020), but a lack of effects of radiation exposure on physiological biomarkers in Chornobyl tree frogs (Burraco, Bonzom, et al., 2021). Here, we examined the differences in dorsal skin coloration of Eastern tree frogs (*Hyla orientalis*) living across a large gradient of radioactive contamination around Chornobyl, and whether the maintenance of skin coloration is physiologically costly in terms of oxidative stress.

Based on the putative protective role of melanin against radiation, we predicted that frogs living in (or near) areas with high radiation levels would present a darker skin coloration, which may suggest that radiation acted as a selective pressure on that trait. Since coloration is known to quickly change in some amphibians during the breeding season (Hettyey et al., 2009; Nilsson Sköld et al., 2013), we empirically examined the short‐term lability of coloration through a small laboratory‐based experiment. Finally, we explored the links between coloration, radiation and oxidative stress. Ionizing radiation is predicted to induce an oxidative stress state in cells (Einor et al., 2016; Galván et al., 2014), a process that may be buffered by the production of melanin or accentuated by the physiological costs of producing pigmentation. However, as eumelanin is the main pigment involved in the production of dark coloration in frogs, and a few studies support that its production seems not to be oxidatively costly (Frost‐Mason & Mason, 1996; Galván et al., 2011; Prota, 1992), we predicted that darker frogs would not experience significant oxidative stress.

## 2 MATERIAL AND METHODS

### 2.1 Field sampling

We examined skin coloration on the Eastern tree frog (*Hyla orientalis*), a cryptic species of the European tree frog (*Hyla arborea*) group that inhabits from the Caspian to the Baltic Sea. Females start to breed at 2–3 years of age (Özdemir et al., 2012), which means that 10–15 generations have passed since the Chornobyl accident. The species usually presents a light green coloration (https://amphibiaweb.org/species/7317).

**FIGURE 1:**
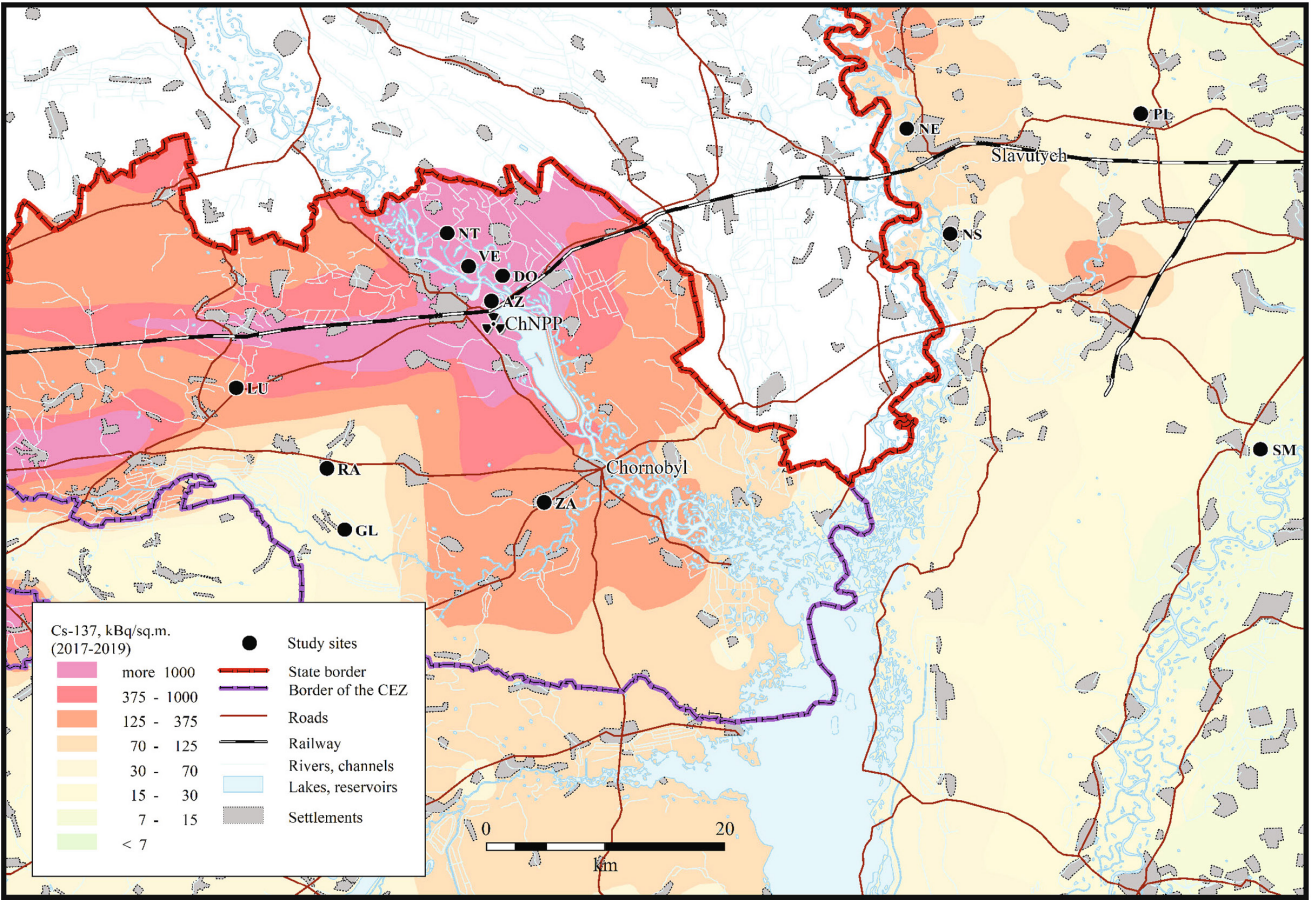
Map of the studied eastern tree frog (*Hyla orientalis*) locations (see also Table S1). The underlying ^137^Cs soil data (decay corrected to spring 2017–2019) is derived from the atlas of radioactive contamination of Ukraine (Intelligence Systems GEO, 2011).

During three consecutive breeding seasons (2017–2019), we collected reproductive adult males of *H. orientalis* in ponds located in a 2.200 km^2^ area around Chornobyl (Northern Ukraine, Figure 1; Supporting Information). We sampled frogs in twelve localities: eight within the Chornobyl Exclusion Zone and four outside Chornobyl in an area situated ca. 40 km to the East of the Exclusion Zone and currently exposed only to background radiation levels (Figure 1; Table S1). These two areas are separated by a system of flood plains, channels, and rivers (maximum crossing distance for a frog ca. 100 m) and moist soils (see USDA Foreign Agricultural Service https://ipad.fas.usda.gov/cropexplorer/imageview.aspx?regionid=umb; or Copernicus Soil Moisture Index: https://land.copernicus.eu/global/products/ssm). Both areas are located within the Polesia woodland ecological zone biome (Fileccia et al., 2014), and our previous studies have revealed no geographic barriers, and the existence of gene flow, between *H. orientalis* inhabiting localities within these two areas (Car et al., 2022). Field and laboratory work were designed to reduce differences in handling time, temperature or experimental procedures between localities and areas. All localities were medium‐small wetlands with reed beds, within a matrix of forest and meadows, and located within the same soil type and color: soddy‐podzolized sandy and clay‐sandy soils (Soil Map of Ukraine, https://esdac.jrc.ec.europa.eu/content/title‐russia‐soil‐map‐ukraine). Water pH values, which may be associated with changes in coloration (Ancans et al., 2001), did not differ between localities within and outside the Chornobyl Exclusion Zone (average ± SE: 6.9 ± 0.17 for localities within Chornobyl, 7.2 ± 0.24 for localities outside Chornobyl; *χ* ^2^_[1,12]_ = 1.66, *p* = 0.226). Temperature during the sampling days did not differ between localities situated within and outside the Chornobyl Exclusion Zone for both daily maximum and minimum values (average ± SE: 20.0 ± 1.0 and 9.3 °C ± 0.9, respectively, for localities within Chornobyl; 22.5 ± 1.6 and 12.2 ± 1.5 °C, respectively, for localities outside Chornobyl; *χ* ^2^(1, 12) = 1.77, *p* = 0.210 and *χ* ^2^(1, 12) = 2.60, *p* = 0.133 for maximum and minimum temperature, respectively).

In total, we examined 189 *H. orientalis* adult males, captured during active calling from 10 PM to 1 AM. Once captured, we placed frogs in plastic bags and transported them to the laboratory where we stored them individually in small plastic buckets with perforated lids, containing ca. 3 cm of water, and kept them in darkness during the rest of the night. On the next morning, we took a photograph of each individual for colorimetric evaluation (see below), we recorded morphological measurements (snout‐to‐vent length, body depth and width) using a calliper to the nearest 1 mm, and weighted each individual using a precision balance to the nearest 0.01 g. Morphometric measurements were used to estimate body condition index and individual dose rates (see below). All animals were collected, and experimental procedures were conducted, under permit of Ministry of Ecology and Natural Resources of Ukraine (No. 517, 21.04.2016).

### 2.2 Skin colour measurements

We quantified dorsal skin coloration of tree frogs following Troscianko and Stevens (2015). Individuals were placed on a black background and beside a ColorChecker passport (X‐Rite, Inc). To provide a uniform light spectrum, we used two LED lamps (Northlight Isac 5 W, 300 lm, 3000 K), placing one at each side of the frog. We took dorsal images of individuals placed in this setting using a Fuji XT‐1 digital camera with a Fujinon XF 35 mm F2 R WR lens, tripod‐mounted and provided with a Fujifilm RR‐100 remote release. We manually set lens aperture to f5.6, and ISO to 1250. All images were saved as. RAF, a file format containing fully uncompressed photography data. We linearized images with the help of a ColorChecker chart, and using the *Multispectral Image Calibration and Analysis Toolbox* for ImageJ software (Mica toolbox version 2.2.2) developed by Troscianko and Stevens (2015). Once completed the linearization process, we transformed images to greyscale and selected five squares of ca. 4 mm^2^ along the dorsal skin of frogs (two in the front part, two in the mid‐lateral part, and one in the back of the body) avoiding areas where light was reflected directly back (i.e. specular reflectance). We quantified dorsal coloration as luminance using the function *Pattern Color and Luminance Measurements* included in the *Mica toolbox* ImageJ plug‐in (Troscianko & Stevens, 2015). Luminance is an integrative trait that specifically describes the perceived lightness of an individual (see e.g. Carter et al., 2020). Luminance values were scaled to a range of 0–100% in reflectance, corresponding to the black and white luminance standards, respectively. For each individual, we calculated a single value for dorsal skin luminance, as the average luminance of the five selected areas.

### 2.3 Skin colour lability

Male frog coloration can change quickly in function of intrinsic and extrinsic factors such as the intensity of male calling activity or in response to some environmental conditions (e.g. Hettyey et al., 2009; Nilsson Sköld et al., 2013). To determine whether tree frog skin luminance can change over a short time period and how it may be affected by environmental background coloration, in May 2019, we collected 14 *H. orientalis* males from a single location within the Chornobyl Exclusion Zone (Azbuchyn, AZ, one of the localities with the highest variation in luminance; Figure 1) and kept them under contrasting experimental backgrounds. We transported the frogs to the laboratory and maintained them for 48 h into 1‐L plastic buckets with the walls covered with either a white (*n* = 7) or black (*n* = 7) plastic film (except the lid that was transparent and perforated to allow light and air to pass through) to simulate extreme background conditions. Buckets contained ca. 3 cm of water in order to maintain moisture. We photographed each individual at the start and at the end (48‐h later) of the experiment, and dorsal skin luminance was quantified as indicated above.

### 2.4 Oxidative stress

We determined the activity of three antioxidant enzymes (catalase, CAT; glutathione reductase, GR; and glutathione peroxidase, GPX) and lipid peroxidation (malondialdehyde concentration, MDA) in the liver of *H. orientalis* males inhabiting inside the Chornobyl Exclusion Zone. Antioxidant enzymes are present in different cellular compartments and have an essential role in transforming reactive oxygen substances produced during catabolism into more stable and non‐toxic molecules (Halliwell & Gutteridge, 2015). MDA is the main product formed during the peroxidation of membrane polyunsaturated fatty acids and is considered a marker of oxidative damage in lipids (Del Rio et al., 2005).

To explore the relationship between the frog redox status, skin coloration and radiation, we quantified oxidative stress parameters in individuals collected within the Chornobyl Exclusion Zone in 2018. Due to logistical limitations, we did not quantify oxidative levels in individuals inhabiting outside the Chornobyl Exclusion Zone. For the relationship between oxidative stress and coloration, high luminance values (i.e. brighter frogs, the most common coloration of the species) were considered as a reference. For the relationship between oxidative stress and radiation, individuals with low absorbed dose rates were considered as the reference ones, following the approach used in previous radioecological research in Chornobyl (e.g. Mappes et al., 2019; Webster et al., 2016). Once morphometric measurements were recorded, we euthanized frogs by pithing without decapitation (AVMA, 2020). We collected the liver of each individual and stored them at −80°C until assayed. Livers were immersed in a buffered solution (Burraco et al., 2017) to avoid proteolysis in a proportion tissue:solution of 1:4.5, and homogenized at 35,000 rpm. The homogenates were centrifuged at 20,817 *g* for 30 min at 4°C, and the supernatants were used to estimate levels of the oxidative stress markers. As the activity of each antioxidant enzyme is relative to the amount of protein contained in the sample, we first determined total protein content according to Bradford's procedure (Bradford, 1976). We quantified catalase activity according to Cohen and Somersonm (1969). This method uses potassium permanganate (KMnO_4_) as an oxidizing agent and coloured component of the reducing agent H_2_O_2_. Five minutes after adding KMnO_4_, we quantified the reduction in this compound at a wavelength of 490 nm. We quantified glutathione peroxidase and glutathione reductase following Paglia and Valentine (1967) and Cribb et al. (1989), respectively, measuring in both cases NADPH oxidation at a wavelength of 340 nm. Finally, we quantified malondialdehyde (MDA) by measuring the red colour formed after the reaction of MDA with thiobarbituric acids, at a wavelength of 535 nm and according to Buege and Aust (1978).

### 2.5 Exposure to radiation

To estimate current exposure to radiation, we quantified total dose rates absorbed by each frog (in μGy/h). Briefly, we estimated total activity of ^90^Sr and ^137^Cs by integrating radionuclide measurements with body mass of each individual, considering the relative mass of bones (10%) and muscles (69%). We combined radionuclide activity concentrations in frogs, soil, and water with dose coefficients (in μGy/h per Bq per unit of mass). Dose coefficients for *H. orientalis* were calculated for internal and external exposure using a theoretical habitat use scenario for the species during the breeding season (Burraco, Car, et al., 2021) with the help of EDEN v3 IRSN software (Beaugelin‐Seiller et al., 2006). For each frog, total individual dose rate was calculated by summing internal and external dose rates (see full details in Burraco, Car, et al., 2021). To estimate historical exposure to radiation in the studied localities, we calculated the distance from each place to the closest area with historical high radiation level, using as reference areas with ^137^Cs contamination levels above 3700 kBq/m^2^ in May 1986 (Cort et al., 2009).

### 2.6 Statistical analyses

All analyses were conducted in R version 3.6.1. We explored the relationship between historical and current radiation levels on dorsal skin luminance in tree frogs. We first measured the distance of sampling locations to the closest historical high radiation area (^137^Cs levels >3700 kBq/m^2^ in May 1986) using the maps of the *Atlas of caesium deposition on Europe after the Chernobyl accident* (Cort et al., 2009). As distance of sampling locations to the closest historical high radiation area and current individual dose rates were highly correlated (*R* ^2^ = 0.86), we conducted a linear regression between *luminance* and *distance to the closest historical high radiation area* and *individual dose rate*, including *body condition* as a covariate and *locality* as a random factor. We also conducted a linear regression between luminance and *individual dose rate* in individuals inhabiting the Chornobyl Exclusion Zone. Finally, to examine possible differences between sampling areas (i.e. *Chornobyl Exclusion Zone* and *Outside Chornobyl*) on the dorsal skin luminance of tree frogs, we ran a linear mixed model including *luminance* as the dependent variable, *sampling area* as the independent variable, *body condition* as a covariate, and *sampling location* as a random factor. We added 0.1 unit to each value of *distance to the closest historical high radiation area* and *individual dose rate* value, to avoid 0 values that prevent log transformations. We estimated body condition as the residuals of the regression between snout‐to‐vent‐length and body mass values (Green, 2001).

To check for the possible effect of time and experimental background conditions on short‐term changes in dorsal skin coloration, we ran a linear mixed model including *luminance* as a dependent variable, the interaction between *time* (0 or 48 h) and *background* (black or white) as independent variables, *body condition* as a covariate, and *individual* as a random factor accounting for within‐individual measurements. Finally, we conducted linear mixed regressions between dorsal skin luminance or total individual dose rate and each oxidative stress parameter (CAT, GR, GPX, and MDA, all log transformed), including *body condition* as a covariate, and *sampling location* as a random factor. Oxidative stress values were scaled to report comparable estimates in linear regressions. Data were plotted using the function *ggplot* included in the package *ggplot2* (version 3.3.3).

## 3 RESULTS

**FIGURE 2:**
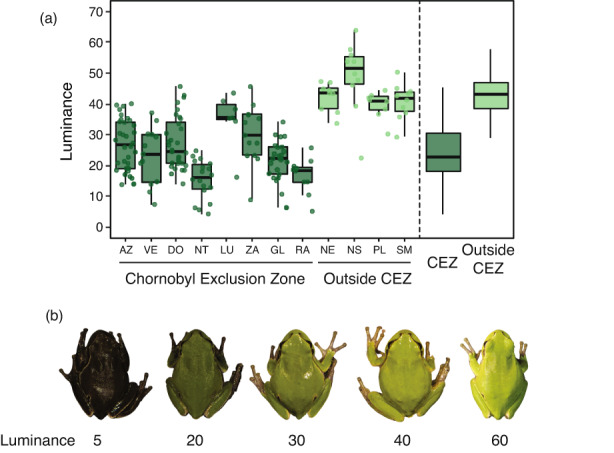
(a) Dorsal skin luminance in eastern tree frog (*Hyla orientalis*) males inhabiting across a gradient of radiation inside (*CEZ*) or outside the Chornobyl exclusion zone (*outside CEZ*). (b) Range of dorsal skin luminance in *H. orientalis* males (from left to right: Luminance values of 5, 20, 30, 40 and 60).

**FIGURE 3:**
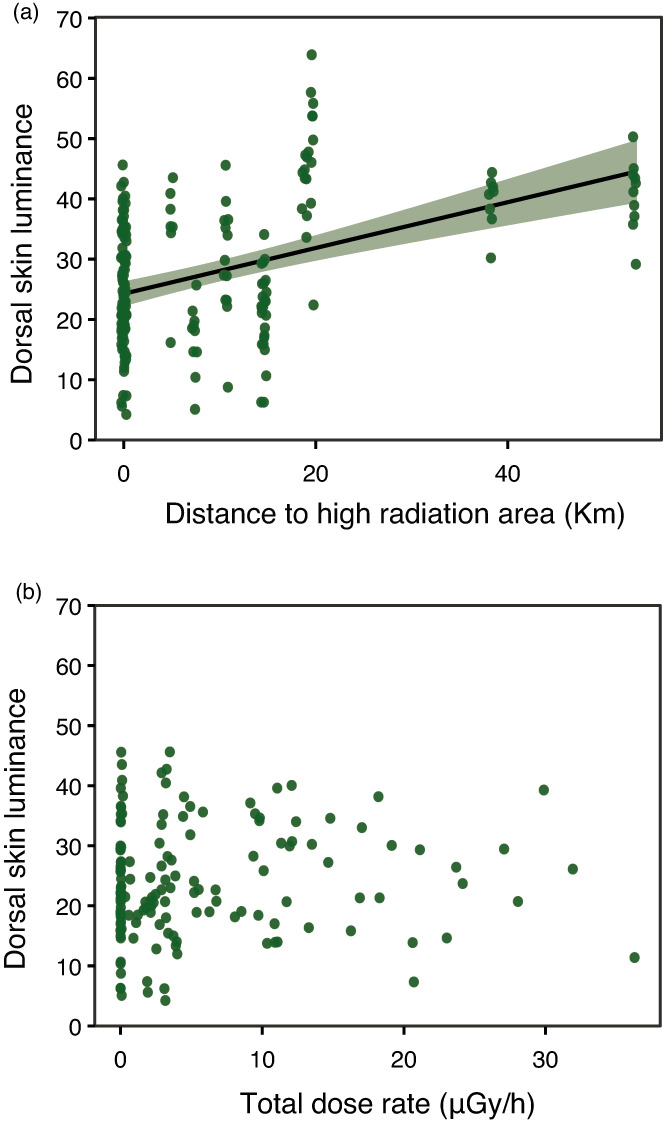
Correlation between dorsal skin luminance in eastern tree frog (*Hyla orientalis*) males and, (a) distance to the closest area with historical high radiation levels (^137^Cs levels >3700 kBq/m^2^ in May 1986), and (b) total individual dose rates of sampled individuals within the Chornobyl Exclusion Zone.

Dorsal skin luminance varied substantially across sampling localities, ranging between 4.2 and 63.9 (4.2–45.6 and 22.4–63.9, within and outside the Chornobyl Exclusion Zone, respectively; Figure 2). In individuals inhabiting within and outside the Chornobyl Exclusion Zone, skin luminance was positively correlated with the distance to the closest area with historical high radiation level (*χ* ^2^_(189)_ = 7.77, *p* = 0.005; Figure 3a), but not to individual dose rate (*χ* ^2^_[189]_ = 2.24, *p* = 0.134). Radiation levels currently experienced by tree frogs living in the Chornobyl Exclusion Zone (i.e. total individual dose rates) were not associated with variation in dorsal skin luminance (*χ* ^2^_[1147]_ = 0.30, *p* = 0.586; Figure 3b). There was no effect of male body condition (proxy for health and fitness in amphibians) on dorsal skin luminance (*p* = 0.26). Overall, Eastern tree frogs (*Hyla orientalis*) living within the Chornobyl Exclusion Zone had 43.6% lower dorsal skin luminance, on average, than frogs living outside Chornobyl (*χ* ^2^_[189]_ = 27.18, *p* < 0.001; Figure 2a,b).

We did not find any correlation between skin luminance and oxidative stress markers (CAT: *χ* ^2^(1, 75) = 0.32, *p* = 0.574; GR: *χ* ^2^ (1, 77) = 1.06, *p* = 0.304; GPX: *χ* ^2^ (1, 78) = 0.76, *p* = 0.382; MDA: *χ* ^2^ (1, 81) = 0.59, *p* = 0.443), but we detected a negative correlation between individual dose rate and lipid peroxidation (i.e. MDA: *χ* ^2^ (1, 81) = 12.36, *p* < 0.001; see Supporting Information). All other correlations between individual dose rates and oxidative stress markers were nonsignificant (all *p* < 0.278). Frog dorsal skin luminance was stable during the 48 h that lasted the experiment on colour lability (*χ* ^2^_[1,14]_ = 0.51, *p* = 0.479) and was unaffected by background colour (*χ* ^2^_[1,14]_ = 0.14, *p* = 0.705). We also did not find a significant interaction between time and experimental background (*χ* ^2^_[1,14]_ = 2.96, *p* = 0.085).

## 4 DISCUSSION

Historical but not current radiation conditions seem to mediate differences in skin coloration in Chornobyl tree frogs. We detected a significant association between dark coloration and proximity to highly contaminated areas in May 1986 (i.e. areas with ^137^Cs levels >3700 kBq/m^2^; Cort et al., 2009). However, we did not detect a significant correlation between individual absorbed dose rates and pigmentation, suggesting that darker coloration is not induced by the current exposure to radiation. On average, tree frogs inhabiting the Chornobyl Exclusion Zone were remarkably darker than individuals from a closely located control area with background radiation levels. Additionally, we found no signs of oxidative costs linked to the maintenance of dark coloration in Chornobyl tree frogs, but lower lipid peroxidation in frogs with high dose rates. Finally, the observed differences in coloration do not seem to be due to short‐term changes in skin coloration, or caused by adjustments to background color. These results suggest that exposure to high levels of ionizing radiation may have selected for dark skin coloration in Chornobyl tree frogs.

Dark melanin‐based pigmentation is known to offer protection against different forms of radiation, including ionizing radiation (Cordero & Casadevall, 2020; Pacelli et al., 2017; Robertson et al., 2012). Exposure of fungi to high levels of ionizing radiation under laboratory conditions generates an increase in melanism and in the production of reactive oxygen species scavenging antioxidant enzymes, suggesting a key protective mechanism of melanin (Kothamasi et al., 2019). Likewise, exposure to ionizing radiation enhances the growth of some melanised fungi (Dadachova et al., 2007), which may even transform ionizing radiation into an energy source through the use of melanin pigments (Dadachova & Casadevall, 2008). Actually, melanised fungi can be found in areas with high natural (i.e. background) radiation such as those exposed to high cosmic radiation at the International Space Station (Dadachova & Casadevall, 2008), and they can even colonize highly radio‐contaminated structures such as the interior of the damaged Chornobyl reactor (Wember & Zhdanova, 2001). Laboratory studies have revealed the putative protective role of melanin against ionizing radiation in vertebrates, although the number of these studies is still very limited and they have been conducted under acute radiation conditions, and thus far from the ones experienced by wildlife in radio‐contaminated areas (e.g. Carotenuto et al., 2016; Kunwar et al., 2012).

Our study suggests that dark coloration in tree frogs is linked to historical but not to current radiation levels. This suggestion is based on a positive correlation between luminance and distance to the highest contaminated area at the moment of the accident, and on the lack of correlation between coloration and current dose rates absorbed by the examined frogs. Radiation levels have dropped several orders of magnitude since the accident and many short‐lived radionuclides known to induce significant biological damage have completely disappeared from the area (e.g. ^131^I; Intelligence Systems GEO, 2011), which can contribute to explain the role of historical exposure and the lack of correlation between luminance and current individual dose rate in our study. Variability in coloration, including dark or grey individuals, is often described in tree frogs of the *Hyla* genus (Wassef et al., 2019). In our study, dark individuals were also detected (although in very low proportions) in localities outside the Chornobyl area (see Figure 2a). It is, thus, plausible that selective processes acting on existing colour variability favoured individuals with darker coloration, linked to higher survival rates in dark frogs under extremely high radiation conditions shortly after the accident. Indeed, pollution can generate strong selective pressures inducing high rates of evolutionary change (Sanderson et al., 2022). In this line, a review by Geras'kin et al. (2008) showed that the severity of the effects of radiation on the physiology or fitness of different species and on the ecosystem structure after the Chornobyl accident was strongly dependent on the dose received in the early period after the accident. This agrees with the idea that historical exposure to radiation may be behind some of the effects detected in the area across taxa, a topic that deserves further research (Beresford et al., 2020; Hancock et al., 2020). If selection acted on frog coloration, low dispersal and high philopatry to the natal environment characteristic of many amphibians (including the study species; Angelone, 2009), may have favoured the prevalence of dark coloration in the Chornobyl tree frog metapopulation (Car et al., 2022). In addition, amphibians with dark skin coloration often show dark coloration in their internal organs (Franco‐Belussi et al., 2016, 2017), and stressful conditions such as oxygen and food deprivation are known to induce higher cellular pigmentation (Franco‐Belussi et al., 2017). Furthermore, although very few studies have examined the heritability of coloration in amphibians, work on different species has revealed that coloration has a genetic basis (Hoffman & Blouin, 2000; Stuckert et al., 2019), a knowledge that can foster the development of further studies investigating the evolutionary causes and consequences behind the observed divergence in coloration in Chornobyl frogs.

In vertebrates, dark coloration is mostly produced by two melanin‐based pigments, black eumelanin and yellow–red pheomelanin. Melanin production is often costly and can generate oxidative stress (in addition to increases in corticosterone levels and metabolic rate; Chang et al., 2021; Polo‐Cavia & Gomez‐Mestre, 2017), which is also one of the main negative effects of the exposure to ionizing radiation (Galván et al., 2014). However, we found no sign of oxidative stress linked to dark coloration in Chornobyl tree frogs, but lower lipid peroxidation in frogs with high dose rates. The latter may be a consequence of compensatory responses, earlier in life, of the oxidative stress machinery against radiation (similar to observed in amphibian larvae in response to early‐life detrimental conditions, e.g. Burraco et al., 2020). In amphibians, dark skin pigments are generated in melanophores, the skin chromatophores responsible for black, brown and darker green coloration (Duellman & Trueb, 1986). Although pheomelanin has been identified in the skin of some frogs (e.g. Wolnicka‐Glubisz et al., 2012), eumelanin seems to be the almost exclusive dark pigment in amphibians (Frost‐Mason & Mason, 1996; Prota, 1992). Eumelanin protects organisms against DNA damage (Galván et al., 2014), and its production may incur in lower costs than pheomelanin, since this pigment is produced without the need of cysteine and GSH (glutathione), a crucial intracellular antioxidant (García‐Borrón & Olivares Sánchez, 2011; Ito et al., 2011). Studies in wild vertebrates exposed to ionizing radiation have reported a strong demographic decline linked to radiation in bird species with pheomelanin‐based coloration, probably due to high consumption of GSH during pheomelanogenesis (e.g. Galván et al., 2011). By contrast, eumelanin levels in feathers were associated with lower oxidative stress and lower DNA damage in birds breeding in the Chornobyl area (Galván et al., 2014). Since oxidative damage is generally high in radio‐contaminated environments (Bonisoli‐Alquati et al., 2010; Einor et al., 2016), the production of eumelanin pigmentation may allow Chornobyl frogs to get protection without incurring in oxidative costs linked to the synthesis of pheomelanin.

Our study design aimed to minimize the effects of other factors potentially affecting coloration, such as environmental characteristics (e.g. habitat and soil type, water pH), capture time, or field and laboratory temperature, and although habitat changes have been substantial in the area, frog breeding habitats have remained much more stable (Santos, 2018). At the species/individual level, melanin‐based coloration can have pleiotropic effects on other traits (San‐Jose & Roulin, 2018). However, in our study, dorsal coloration did not correlate with body condition, a proxy for overall individual performance. Another putative confounding process may be a link between coloration and mating success (observed in some amphibian species, e.g. Rudh et al., 2011), although that seems not to be the case in tree frogs (Gomez et al., 2009). In our laboratory‐based experiment, skin coloration seemed to be stable over a short time period (48 h). In addition, skin coloration was not significantly affected by background coloration, and (even if not significant) those changes were much smaller than the observed differences in skin coloration between frogs inhabiting within and outside the Chornobyl Exclusion Zone. Despite we discarded the possible influence of several confounding factors on the observed coloration pattern, we acknowledge the limitations of our field approach regarding the lack of field‐based experiments and the small sample size used in the experiment testing for lability in coloration. Also, the absence (to the best of our knowledge) of tree frog samples collected in the Chernobyl area before or immediately after the accident, prevents us from comparing current and past skin coloration. Research addressing genome‐wide signatures of selection may also contribute to improving our understanding on the mechanism behind dark coloration in Chornobyl tree frogs.

## 5 CONCLUSIONS

Our results suggest that the protective role of melanin previously detected in Chornobyl in smaller living organisms such as fungi may extend to wild vertebrates exposed to ionizing radiation. Historical high radiation and lack of production costs of eumelanin‐based pigmentation may have facilitated the selection and maintenance of dark coloration in Chornobyl tree frogs. Further studies are needed to disentangle the causes and consequences of darker pigmentation in radio‐contaminated environments, which will help to develop a better understanding of the eco‐evolutionary effects of the long‐term exposure to ionizing radiation on wildlife.

## FUNDING INFORMATION

This work was supported by the Swedish Radiation Protection Agency‐SSM (SSM2018‐2038), by Carl Tryggers Foundation (CT 16:344) and by the Spanish Society of Terrestrial Ecology (AEET). The Carl Tryggers Foundation, Marie Sklodowska‐Curie project METAGE‐797879, and Juan de la Cierva Incorporación (IJC2020‐044680) supported PB, and the Spanish Ministry of Science and Innovation (Ramón y Cajal program, RYC‐2016‐20,656) supported GO.

## CONFLICT OF INTEREST

The authors declare that there is no conflict of interest.

## Supporting information


Table S1
Table S2
Figure S1

## Data Availability

Data associated with this study are made available in the figshare data repository https://doi.org/10.6084/m9.figshare.15124218.v1 Burraco and Orizaola (2022).
