# Supplementary material for: Ionizing radiation and melanism in Chornobyl tree frogs
Source: Evol Appl. 2022 Sep 7;15(9):1469–79. doi: 10.1111/eva.13476 (PMC9488684; doi:10.1111/eva.13476)

**Supplementary Material**

**Table S1** Geographic coordinates (latitude and longitude), number of sampled frogs, current levels of environmental radiation (i.e. ambient dose rate), and area of the locations included in the study. CEZ: Chornobyl Exclusion Zone.

| **Location** | **Code** | **GPS coordinates** | **Sampled**  **frogs (n)** | **Radiation (µSv/h)** | **Area** |  |
| --- | --- | --- | --- | --- | --- | --- |
|  |  |  |  |  |  |  |
|  |  |  |  |  |  |  |
| Vershina | VE | 51.4328, 30.0769 | 13 | 16.20 | CEZ |  |
| Azbuchin | AZ | 51.4047, 30.1044 | 33 | 7.61 | CEZ |  |
| Northern Trace | NT | 51.4567, 30.0486 | 18 | 2.51 | CEZ |  |
| Dolzhikovo | DO | 51.4256, 30.1161 | 32 | 1.50 | CEZ |  |
| Lubianka | LU | 51.3388, 29.7976 | 7 | 0.27 | CEZ |  |
| Zalesie | ZA | 51.2506, 30.1667 | 13 | 0.12 | CEZ |  |
| Glinka | GL | 51.2300, 29.9250 | 25 | 0.10 | CEZ |  |
| Razjezzheie | RA | 51.2786, 29.9050 | 10 | 0.07 | CEZ |  |
| Nedanchichy N | NE | 51.5364, 30.5981 | 10 | 0.08 | Outside CEZ |  |
| Nedanchichy S | NS | 51.4939, 30.6236 | 9 | 0.06 | Outside CEZ |  |
| Smolin | SM | 51.2925, 31.0294 | 10 | 0.04 | Outside CEZ |  |
| Plekhov | PL | 51.5489, 30.8863 | 8 | 0.04 | Outside CEZ |  |
|  |  |  |  |  |  | |

**Table S2** Correlations between dorsal skin luminance of Eastern tree frog (*Hyla orientalis*) males living in the Chornobyl Exclusion Zone, and markers of oxidative stress levels (catalase, CAT; glutathione reductase, GR; glutathione peroxidase, GPX; and malondialdehyde, MDA).

|  |  | | | |  | | |  | | | |  |
| --- | --- | --- | --- | --- | --- | --- | --- | --- | --- | --- | --- | --- |
|  | **Luminance** | | | |  | | | **Total dose rate** | | | |  |
|  |  | | | |  | | |  | | | |  |
|  |  |  |  |  | |  |  | |  |  |  | |
|  | Df | Chi-sq | Estimate | P-value | |  | Df | | Chi-sq | Estimate | P-value | |
|  |  |  |  |  | |  |  | |  |  |  | |
|  |  |  |  |  | |  |  | |  |  |  | |
| **CAT** | 1 | 0.32 | -0.06 | 0.574 | |  | 1 | | 0.22 | 0.06 | 0.639 | |
| **GR** | 1 | 1.06 | -0.12 | 0.304 | |  | 1 | | 0.03 | -0.03 | 0.866 | |
| **GPX** | 1 | 0.76 | 0.10 | 0.382 | |  | 1 | | 2.07 | -0.28 | 0.150 | |
| **MDA** | 1 | 0.59 | 0.09 | 0.443 | |  | 1 | | 12.36 | -0.37 | <0.001 | |
|  |  |  |  |  | |  |  | |  |  |  | |

**Figure S1** Correlation between lipid peroxidation (MDA concentration) and total individual dose rates quantified in males of *H. orientalis* inhabiting Chornobyl Exclusion Zone.


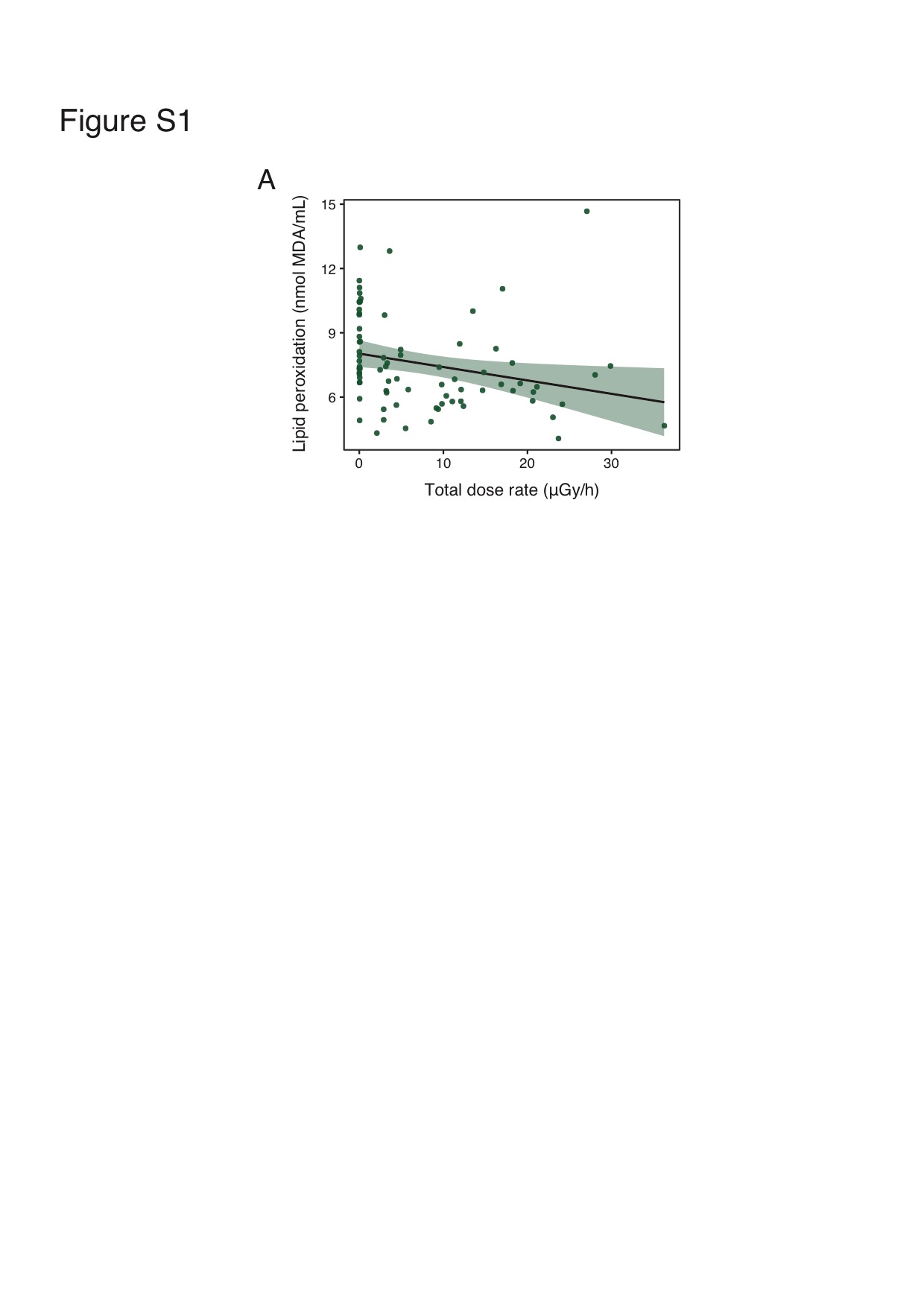

Supplement: Supplementary file 1 — Table S1 Table S2 Figure S1 [file EVA-15-1469-s001.docx]
